# Cost-Effectiveness of Trimodal Therapy and Radical Cystectomy for Muscle-Invasive Bladder Cancer

**DOI:** 10.1001/jamanetworkopen.2025.17056

**Published:** 2025-06-23

**Authors:** Daniel D. Joyce, Kevin M. Wymer, John A. Graves, Stephen A. Boorjian, John L. Gore, Ali Raza Khaki, Ann C. Raldow, Stephen B. Williams, Angela B. Smith, Vidit Sharma

**Affiliations:** 1 Department of Urology, Vanderbilt University Medical Center, Nashville, Tennessee; 2 Department of Urology, Mayo Clinic, Rochester, Minnesota; 3 Department of Health Policy, Vanderbilt University Medical Center, Nashville, Tennessee; 4 Department of Urology, University of Washington School of Medicine, Seattle; 5 Department of Medicine, Division of Oncology, Stanford University, Stanford, California; 6 Department of Radiation Oncology, University of California, Los Angeles; 7 Division of Urology, Department of Surgery, The University of Texas Medical Branch, Galveston; 8 Department of Urology, University of North Carolina at Chapel Hill; 9 Lineberger Comprehensive Cancer Center, University of North Carolina at Chapel Hill

## Abstract

**Importance:**

Trimodal therapy (TMT) is included as an alternative to radical cystectomy (RC) for definitive management of muscle-invasive bladder cancer (MIBC) in current clinical guidelines. Moreover, a 2023 retrospective analysis reported similar oncologic outcomes between these treatments among patients deemed fit for RC. Data regarding the comparative value of these treatments are lacking.

**Objective:**

To evaluate the comparative cost-effectiveness of TMT and RC for treatment of MIBC.

**Design, Setting, and Participants:**

This economic evaluation compared cost-effectiveness of treatments using a health transition state microsimulation model of patients with bladder cancer treated between 2005 and 2017 with 5- and 10-year horizons from a Medicare perspective. Model probabilities were informed by multicenter retrospective analyses published in 2023 comparing TMT with RC. The index patient was aged 71 years, with clinical stage T2-4aN0M0 MIBC, solitary tumor smaller than 7 cm, no or unilateral hydronephrosis, adequate bladder function, and lack of multifocal or extensive carcinoma in situ. Patients unfit for RC, radiation, or cisplatin-based chemotherapy were excluded.

**Exposures:**

TMT and RC.

**Main Outcome and Measures:**

Primary outcomes included effectiveness measured in quality-adjusted life years (QALYs) and incremental cost-effectiveness ratios (ICER) using a willingness-to-pay threshold of $100 000 per QALY. Sensitivity analyses were performed to assess the robustness of the model.

**Results:**

For the index patient, at 5 years, the average cost was $30 525 higher for TMT than RC. Average QALYs were 3.87 and 3.94 for RC and TMT, respectively. As such, TMT was not cost-effective at 5-year (ICER, $464 291 per QALY) or 10-year (ICER, $308 638 per QALY) time horizons. On 1-way sensitivity analyses, TMT would become cost-effective if (1) TMT costs were less than $17 605; or (2) TMT resulted in an 11.6% improvement in metastasis-free survival relative to RC.

**Conclusions and Relevance:**

In this economic evaluation study of TMT and RC for treatment of MIBC, TMT was associated with improved quality of life but was not cost-effective relative to RC at 5 and 10 years given higher treatment costs. These findings highlight the importance of developing policy initiatives that help reduce TMT costs and of providing patients with accurate expectations of long-term toxic effects to help guide preference-sensitive care.

## Key Points

**Question:**

What is the more cost-effective strategy for management of muscle-invasive bladder cancer?

**Findings:**

In this economic evaluation, trimodal therapy was associated with improved quality of life, but was not a cost-effective strategy for management of muscle-invasive bladder cancer relative to radical cystectomy.

**Meaning:**

Policy initiatives are needed to reduce the cost of trimodal therapy, and discussions about long-term tradeoffs of toxic effects between radical cystectomy and trimodal therapy are critical to guide preference-sensitive care.

## Teaser

This economic evaluation compares the cost-effectiveness of treatment and disease management strategies for muscle-invasive bladder cancer from a US payer perspective.

## Introduction

Radical cystectomy (RC) has been utilized in the treatment of muscle-invasive bladder cancer (MIBC) for decades^1,2,3^ and, together with neoadjuvant chemotherapy (in eligible patients), continues to represent standard treatment in current guidelines.^4,5,6^ However, patients with bladder cancer are frequently older and with multiple comorbid conditions.^7^ Moreover, surgical extirpation is associated with significant risks of perioperative complications and even mortality.^3,8^ Despite advancements with orthotopic neobladder reconstruction for urinary diversion, the quality-of-life (QOL) implications of surgery remain a significant concern to many patients and have fueled interest in bladder preservation strategies.

Trimodal therapy (TMT), which involves maximal transurethral resection of bladder tumor (TURBT) followed by chemotherapy and radiation, has become established as an alternative management for patients with MIBC who are unable or unwilling to undergo RC. TMT affords bladder preservation, avoids the morbidity associated with surgery, and may thereby be associated with improved QOL. Indeed, guidelines now include TMT as an option for patients with MIBC.^4,5,6^ Relative oncologic efficacy of these approaches remains in question given the lack of randomized trials comparing TMT with RC. Prior efforts at such trials have been unsuccessful due to poor accrual, and future similar studies are unlikely.^9^ Therefore, evidence regarding the comparative effectiveness of these treatment options is limited to retrospective analyses. Of note, data from a large, 2023 multicenter retrospective cohort study suggested similar cancer-specific (CSS) and overall survival (OS) between RC and TMT among patients who were eligible for either approach.^10^

Of further importance to investigate is the relative cost of care of these treatments to the health care system. Cost of care is of particular concern in the setting of bladder cancer, which is associated with a lifetime cost of $96 000 to $187 000 per diagnosis, making it one of the costliest malignant neoplasms to treat.^11,12,13^

Currently, there are limited data regarding the cost and utility tradeoffs between RC and TMT. Herein, therefore, we sought to evaluate the comparative cost-effectiveness of these treatments using a microsimulation model, accounting for potential downstream costs and toxic effects, from a US health care perspective.

## Methods

### Patient Population

This study was a cost-effectiveness analysis comparing TMT with RC for treatment of MIBC. The index patient utilized for analysis reflects those included in the 2023 multicenter retrospective study by Zlotta et al^10^: age 71 years, clinical stage T2-4aN0M0 muscle-invasive urothelial carcinoma treated between 2005 and 2017, solitary tumor of a size less than 7 cm, no or unilateral hydronephrosis, adequate bladder function, and lack of multifocal or extensive carcinoma in situ. Importantly, patients unfit for radical cystectomy, radiation, or cisplatin-based chemotherapy were excluded.

This study was exempt from institutional review board review per the Mayo Clinic of Rochester, Minnesota. Findings are reported in concordance with the Consolidated Health Economic Evaluation Reporting Standards (CHEERS) reporting guideline.

### Microsimulation Model Structure

Using TreeAge Pro (Healthcare Version) version 2021, we created a microsimulation model comparing costs and utilities between TMT and RC. TMT consisted of 2 TURBTs to achieve maximal resection as well as single-agent cisplatin radiosensitizing chemotherapy administered weekly at a dose of 40 mg/m^2^ prior to the administration of radiotherapy for 6 total doses.^14^ An average body surface area of 1.79 m^2^ was used to calculate the total dose. Patients underwent computed tomography simulation followed by 3-dimensional–conformal radiation therapy. Bladder-only radiotherapy was administered using a 4-field technique on a linear accelerator. A total dose of 64 Gy was delivered in 32 fractions using 6- to 15-MV photons over a period of 6.5 weeks.^15^

Radical cystectomy included urinary diversion, bilateral pelvic lymph node dissection, removal of both the bladder and prostate in men, and removal of the anterior vagina, uterus, and bilateral ovaries in women. Receipt of neoadjuvant chemotherapy was assumed to be equivalent between treatment arms and was, therefore, not included in the model.^16^ This assumption was consistent with the findings from Zlotta et al,^10^ in which receipt of neoadjuvant or adjuvant chemotherapy were similar between treatment groups (RC, 59% vs TMT, 56%).

The TMT treatment group was modeled with the following health states: no progression (surveillance), progression to metastasis, recurrent MIBC, recurrent non-MIBC (NMIBC), progression after first-line treatment of metastatic disease, progression after second-line treatment of metastatic disease, short-term treatment toxic effects, long-term genitourinary and gastrointestinal toxic effects, and death (eFigure 1 in Supplement 1). Progressions to MIBC, NMIBC, and metastases were treated with salvage RC, induction intravesical BCG, and gemcitabine and cisplatin chemotherapy, respectively.^17^ Second progression among those being treated for metastatic disease was treated with pembrolizumab.^18^ Radical cystectomy health states consisted of no progression (surveillance), progression to metastasis, second progression after treatment of metastatic disease, short-term (perioperative) toxic effects, long-term toxic effects, and death. Long-term toxic effects were categorized as bowel (small bowel obstruction), para-stomal hernia, and uretero-enteric anastomotic complications. There is a lack of evidence describing infectious and stone-related complications following TMT, and so the probability of these complications was assumed to be equivalent between TMT and RC and was, therefore, excluded from the model (eAppendix in Supplement 1).

A time cycle of 3 months was used. A 5-year time horizon was chosen for the base case analysis given the follow-up period of retrospective data, advanced age of the index patient, and uncertainty of long-term complications associated with TMT. As a sensitivity analysis, a 10-year time horizon was also assessed to more closely reflect a lifetime horizon in this cohort and understand potential implications of toxic effects uncertainty on longer-term outcomes.

### Statistical Analysis

#### Probability and Utility Values

Probabilities of progression, CSS, and OS were derived from the multicenter retrospective study by Zlotta et al.^10^ Because analyses performed in this study found no statistically significant differences in progression-free survival and CSS, these probabilities were treated as equivalent in our model. The probability of progression to MIBC requiring salvage cystectomy in the TMT treatment group was also derived from the multicenter retrospective data (13% at 5 years).^10^ We assumed these instances were due to disease progression and not radiation toxic effects resulting in end-stage bladder. As such, no simulated patients received salvage cystectomy for severe treatment toxic effects. All other probabilities were obtained from prior research (eTable 1 in Supplement 1).

#### Costs and Utility Values

Costs included in the model were estimated from a Medicare payer’s perspective adjusted to 2021 US dollars (eTable 2 in Supplement 1). Utility values, ranging from 0 to 1 for each health state, were obtained from prior research (eTable 3 in Supplement 1). Utility values, tolls, and time spent within a given health state were used to calculate quality adjusted life years (QALYs). Future costs and QALYs were annually discounted at a rate of 3% to account for the time value of money and health benefits.

#### Outcomes

Comparative effectiveness (measured in QALYs), costs (in US dollars), and incremental cost-effectiveness ratio (ICER) between treatment groups were the primary outcomes of interest. A willingness to pay (WTP) threshold of $100 000 per QALY was used to determine whether a treatment was cost-effective from US health care systems perspective.^19^

#### Sensitivity Analyses

One-way sensitivity analyses were performed for each variable to better understand how uncertainty influenced outcomes. Two-way sensitivity analyses were then performed to evaluate how simultaneous changes in comparative treatment costs, acute toxic effects, and long-term toxic effects altered the cost-effectiveness of each treatment option. Finally, probabilistic sensitivity analyses using 100 000 Monte Carlo simulations were used to assess the robustness of the model. This number of simulations was chosen to ensure that uncertainty model estimates converge. γ Distributions were used for costs and β distributions were used for probability and utility values.

## Results

### Base Case

The index patient was aged 71 years, with clinical stage T2-4aN0M0 MIBC, solitary tumor smaller than 7 cm, no or unilateral hydronephrosis, adequate bladder function, and lack of multifocal or extensive carcinoma in situ. At a 5-year time horizon, the overall cost of RC was less than TMT ($40 489 vs $71 014, respectively). However, TMT was associated with improved QOL compared with RC (3.94 vs 3.87 QALYs, respectively) due to less accrued disutility from acute and chronic toxic effects. Nevertheless, these advantages in QOL were outweighed by the increase in costs, and as such we found that TMT was not cost-effective relative to RC with an ICER of $464 291 per QALY (Table). Distributions of incremental costs and effectiveness at 5 years are shown in eFigure 2 in Supplement 1.

**Table.** Cost-Effectiveness Analysis Results

Variable	Cost, $	QALY	ICER, $/QALY^a^
**5 y**			
Radical cystectomy	40 489	3.87	1 [Reference]
Trimodal therapy	71 014	3.94	464 291
**10 y**			
Radical cystectomy	49 570	6.49	1 [Reference]
Trimodal therapy	85 137	6.61	308 638

Abbreviations: ICER, incremental cost-effectiveness ratio; QALY, quality-adjusted life year.

^a^ An ICER of less than $100 000 (willingness to pay threshold) was considered cost-effective.

### Extrapolation to 10-Year Time Horizon

At a 10-year time horizon, TMT remained more costly ($85 137 vs $49 570) and, reflecting the improved QOL, more effective (6.61 vs. 6.49 QALYs) compared with RC (Table). However, despite superiority in QOL, with the increased costs of TMT, RC remained the most cost-effective option (ICER for TMT, $308 636 per QALY). The distributions of incremental costs and effectiveness at 10 years are shown in eFigure 3 in Supplement 1.

### One-Way Sensitivity Analyses

**Figure 1.**
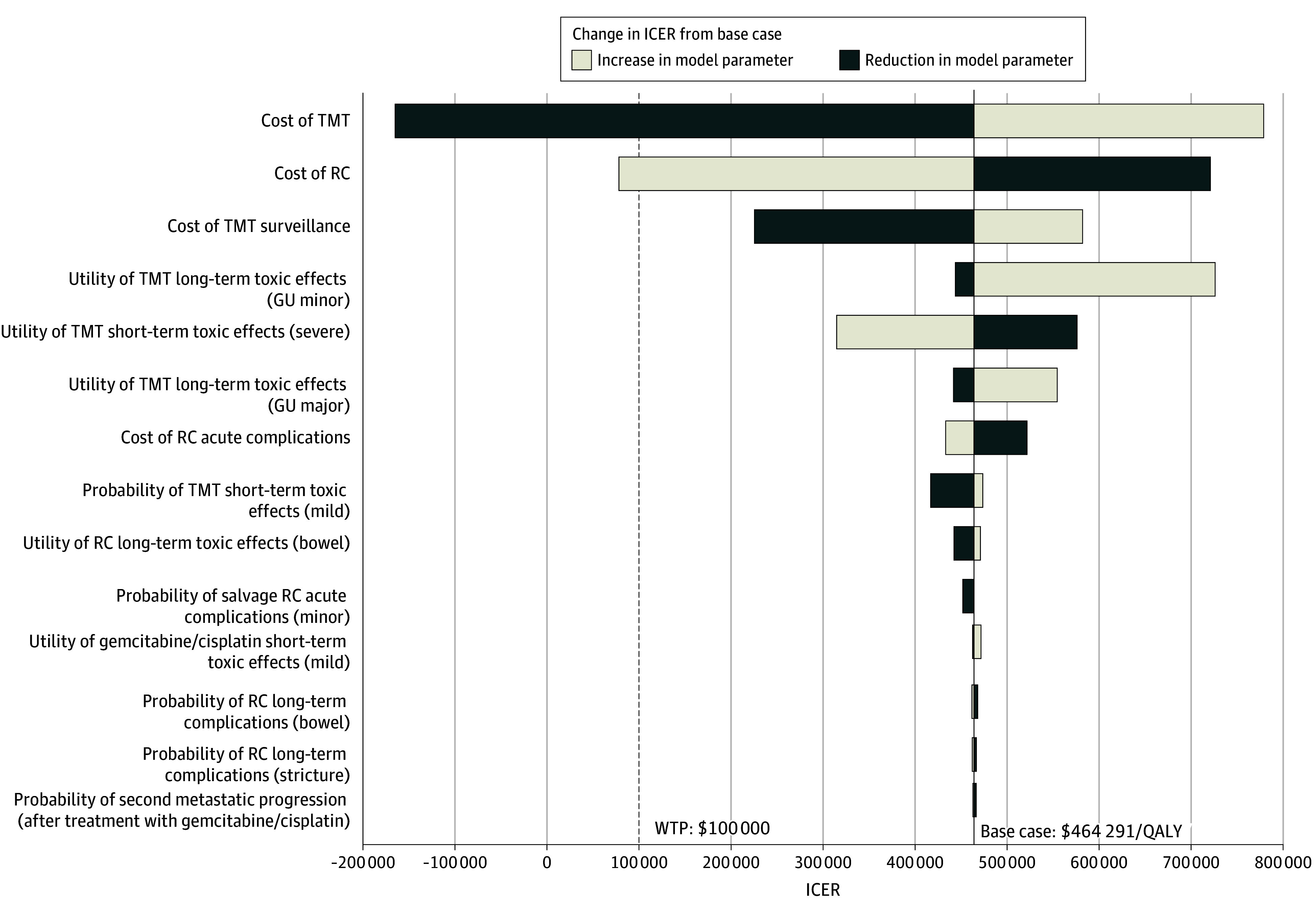
Tornado Diagram of ICER 1-Way Sensitivity to Model Parameters GU indicates genitourinary; ICER, incremental cost-effectiveness ratio; QALY, quality-adjusted life year; RC, radical cystectomy; TMT, trimodal therapy.

Model outcomes were most sensitive to costs of initial treatment and disutility from long-term toxic effects (Figure 1). For example, if the initial cost of TMT was reduced to $17 605 (base case, $41 777), it would become the more cost-effective option. Similarly, if the initial cost of RC increased to $48 129 (base case, $19 923), TMT would be the more cost-effective option. Alternatively, if current prices are maintained, TMT would need an absolute metastasis risk reduction of 11.6% to become cost-effective (with base case metastasis difference equalling 0%).

### Two-Way Sensitivity Analyses

**Figure 2.**
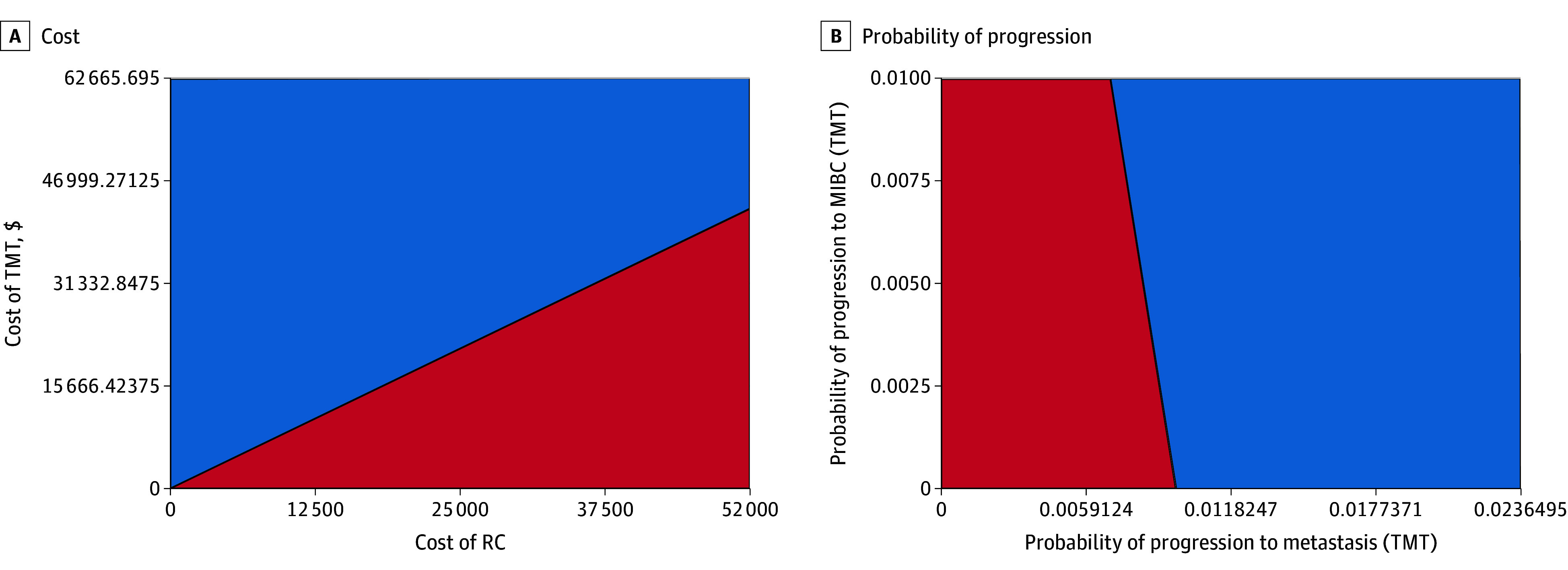
Two-Way Sensitivity Analyses Comparing Costs and Probability of Progression for RC vs TMT RC indicates radical cystectomy; MIBC, muscle-invasive bladder cancer; TMT, trimodal therapy. Blue represents combinations in which RC is cost effective and red represents combinations in which TMT is cost effective.

Multiple combinations of model variables were explored in 2-way sensitivity analyses to better understand the impact of aggregate toxic effects on model outcomes. These analyses were unable to identify a combination of toxic effects that resulted in TMT becoming the more cost-effective approach. Treatment costs and probabilities of progression to metastatic disease continued to have the most impact on model outcomes. Indeed, progression to metastatic disease had greater impact on model outcomes compared with recurrence of MIBC among patients managed with TMT, suggesting that the incidence of salvage cystectomy in these patients had minimal impact on costs over time (Figure 2).

### Probabilistic Sensitivity Analysis

**Figure 3.**
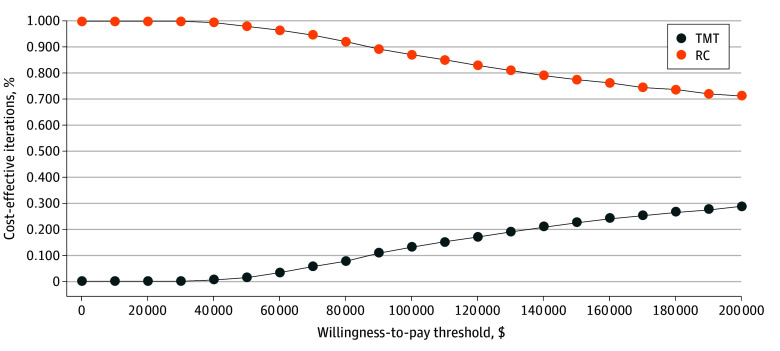
Cost-Effectiveness Acceptability Curve at a 5-Year Time Horizon RC indicates radical cystectomy; TMT, trimodal therapy.

**Figure 4.**
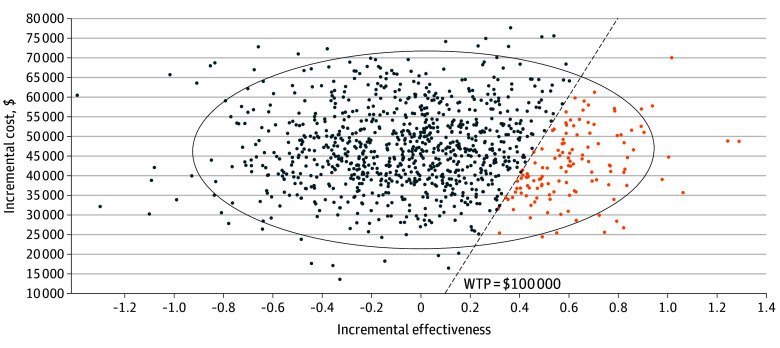
Cost-Effectiveness Scatterplot Each point represents a simulated outcome on probablistic sensitivity analysis—orange dots indicate simulations in which trimodal therapy was the most cost-effective option; blue dots, simulations for which radical cystectomy was the most cost-effective option. The willingness-to-pay threshold was set at $100 000/quality-adjusted life-year. The 95% CI for all simulation outcomes is represented by the ellipse.

Using a willingness-to-pay threshold of $100 000 per QALY, Monte Carlo probabilistic sensitivity analysis at a 5-year time horizon found RC and TMT to be cost-effective in 87% and 13% of microsimulations, respectively (Figure 3). The cost-effectiveness estimate uncertainty is represented in an incremental cost-effectiveness scatterplot, including the 95% CI ellipse and proportion of simulations in which TMT was considered cost-effective (Figure 4). At a 10-year time horizon, RC and TMT were cost-effective in 79% and 21% of microsimulations, respectively.

## Discussion

Using a microsimulation model accounting for both short-term and long-term toxic effects of treatment over time, we found RC to be more cost-effective than TMT from a US Medicare perspective. Although TMT continued to modestly accrue relatively more QALYs than RC over time, the high costs currently associated with this approach prevented TMT from meeting commonly accepted willingness-to-pay thresholds in the US health care system. Nevertheless, our microsimulation model did note that TMT would be the most cost-effective choice for 21% of index patients over 10 years, supporting its use in some scenarios for appropriately counseled patients. These findings highlight the importance of addressing rising costs of bladder cancer care through health policy initiatives and further emphasize the need for ongoing work to better characterize QOL and oncologic outcome differences between RC and TMT to guide preference-sensitive care.

The lack of prospective clinical trial evidence has created a barrier to informed patient counseling regarding the optimal management of MIBC. In 2007, the SPARE trial attempted to compare TMT with RC in patients fit for RC through a randomized clinical trial.^9^ However, strong aversion to undergo randomization among both clinicians and patients led to poor accrual and premature trial closure. As such, estimates of the comparative effectiveness of TMT vs RC remain dependent on and limited by observational data. In a 2019 systematic review and meta-analysis,^20^ pooled analyses found that RC was associated with improved CSS and OS compared with TMT. However, these findings were brought into question following the 2023 publication of the largest TMT multicenter retrospective cohort to date.^10^ In this study, patients with TMT were matched 3:1 to patients with RC, and rigorous statistical methods were employed to attempt to account for potential bias and confounding. The results showed no statistically significant difference in metastasis-free survival (MFS) or OS. Importantly, our model was sensitive to these outcomes, stressing the need to improve our understanding of the comparative oncologic efficacy between TMT and RC.

Reliance on multiple specialized services administering ongoing treatments for an extended period not surprisingly results in higher health care costs with TMT noted here. Indeed, a previous assessment of long-term costs of MIBC management using the Surveillance, Epidemiology, and End Results Program Medicare database revealed that TMT was significantly more expensive than RC at 2 years, and that the gap in cost continued to increase over 5-year follow-up ($424 570 vs $253 651; median difference, $124 466). Notably, these costs are much higher than those described in our analysis.^21,22^ Interestingly, a Canadian single center retrospective study was more consistent with the overall cost values included in our model.^23^ However, unlike our study, costs were higher for RC compared with TMT during the treatment phase, while the cost of follow-up care was higher for TMT (although this result was not significant).^23^ Such analyses may offer insight into the potential for policy interventions to impact cost-effectiveness of MIBC treatments. For example, in a recent cost-effectiveness analysis of TMT and RC from a Canadian health system perspective, mean costs of RC and TMT at 5 years were $29 992 and $30 266 (approximate US dollar equivalent, $21 995 vs $22 193), respectively.^24^ With this minimal difference in costs, TMT was indeed cost-effective at a 10-year horizon with an ICER of $19 746 per QALY (US equivalent, $14 270 per QALY). This stark contrast suggests that price interventions can drastically improve the value of MIBC treatment and should be further explored.

While evaluating cost-effectiveness may help inform the impact of treatments from a health care perspective, to individual patients value is highly dependent on the relative QOL associated with treatment. In fact, Magee et al^25^ demonstrated a higher quality-adjusted life expectancy with TMT compared with RC, while a 2024 meta-analysis^26^ similarly suggested that TMT may be superior to RC regardless of the type of urinary diversion chosen. On the other hand, Chaballout et al^27^ developed utility values using both a visual analogue scale and a standard gamble approach for 6 hypothetical MIBC treatment–related health states. Interestingly, observed utilities were similar between RC with ileal conduit, RC with neobladder, and TMT regardless of which methodology was used. Our model supports claims of slightly superior QOL associated with TMT that continues to rise modestly over time. Given the small magnitude of this observed difference in QOL, our data highlight the need for more rigorous prospective measurements of long-term QOL outcomes after both RC and TMT. Furthermore, comparative patient out-of-pocket costs and indirect costs from these treatments are largely unknown and likely have important influence on QOL outcomes as well.^28^

### Limitations

This study had several limitations. As with any cost-utility analysis, the findings are subject to the limitations of the assumptions used to create the model. That is, our findings are limited to the selection bias inherent in the retrospective studies that were used to garner these values. Model inputs were reliant on the highest quality evidence available for comparative oncologic and QOL outcomes. Threshold values at which point the model outcome would change are similarly subject to these limitations. We have provided distributions for incremental costs and effectiveness for the base case to better clarify the level uncertainty present in these outcomes (eFigure 2, eFigure 3 in Supplement 1). All NMIBC recurrences after TMT were treated with BCG, which may overestimate the cost by not accounting for a proportion of patients that may have qualified for intravesical chemotherapy regimens. Moreover, our model was designed and analyzed prior to the publication of findings from the EV-302/KN-A39 trial,^29^ which showed significant progression free and OS advantages compared with chemotherapy. As such, the impact of these treatments on efficacy and cost were not evaluated in our study. However, given the assumed equivalent MFS between treatments, inclusion of these higher cost combination treatments would likely widen the necessary MFS difference required for TMT to be the most cost-effective option. Likewise, while our model did not include systemic therapy regimens which would be used for patients deemed cisplatin ineligible, inclusion of such treatments would be unlikely to alter model outcomes. Finally, our model assumes that all patients treated with TMT received the same treatment regimen, which is unlikely in real world practice. Practice variation in this space likely influences the value of TMT and was not accounted for in our model. Further work is needed to better understand these variations so that future economic analyses can accurately assess the degree to which they influence health care spending.

## Conclusions

In an analysis with presumed equivalent oncologic efficacy, RC remained the more cost-effective management strategy for patients with MIBC, due primarily to the high costs of treatment associated with TMT. At the same time, TMT was associated with a QOL advantage and was found to be the favored choice for 21% of simulated patients at 10 years. These findings highlight the importance of developing policy initiatives that help reduce TMT costs and of providing patients with accurate expectations of long-term toxic effects to help guide preference-sensitive care.

## Notes

Supplement 1. **eAppendix.** Model Assumptions **eTable 1.** Probabilities **eTable 2.** Costs **eTable 3.** Utilities **eFigure 1.** State Transition Diagram **eFigure 2.** Incremental Cost and Effectiveness Histograms—Distribution of Comparative Outcomes Over 100 000 Microsimulations at a 5-Year Time Horizon **eFigure 3.** Incremental Cost and Effectiveness Histograms—Distribution of Comparative Outcomes Over 100 000 Microsimulations at a 10-Year Time Horizon
eReferences.

Supplement 2.
Data Sharing Statement
