# Supplementary material for: Cost-Effectiveness of Trimodal Therapy and Radical Cystectomy for Muscle-Invasive Bladder Cancer
Source: JAMA Netw Open. 2025 Jun 23;8(6):e2517056. doi: 10.1001/jamanetworkopen.2025.17056 (PMC12186510; doi:10.1001/jamanetworkopen.2025.17056)
Supplement: Supplement 1. — eAppendix. Model Assumptions eTable 1. Probabilities eTable 2. Costs eTable 3. Utilities eFigure 1. State Transition Diagram eFigure 2. Incremental Cost and Effectiveness Histograms—Distribution of Comparative Outcomes Over 100 000 Microsimulations at a 5-Year Time Horizon eFigure 3. Incremental Cost and Effectiveness Histograms—Distribution of Comparative Outcomes Over 100 000 Microsimulations at a 10-Year Time Horizon eReferences. [file jamanetwopen-e2517056-s001.pdf]

# Supplemental Online Content

Joyce DD, Wymer KM, Graves JA, et al. Cost-effectiveness of trimodal therapy and radical cystectomy for muscle-invasive bladder cancer. *JAMA Netw Open.* 2025;8(6):e2517056. doi:10.1001/jamanetworkopen.2025.17056

**eAppendix.** Model Assumptions

**eTable 1.** Probabilities

**eTable 2.** Costs

**eTable 3.** Utilities

**eFigure 1.** State Transition Diagram

**eFigure 2.** Incremental Cost and Effectiveness Histograms—Distribution of Comparative Outcomes Over 100 000 Microsimulations at a 5-Year Time Horizon

**eFigure 3.** Incremental Cost and Effectiveness Histograms—Distribution of Comparative Outcomes Over 100 000 Microsimulations at a 10-Year Time Horizon

**eReferences.**

© 2025 Joyce DD et al. *JAMA Network Open*.

**eAppendix. Model Assumptions**

- We assumed that the duration of disutility from MIBC and NMIBC intravesical recurrence among those patients initially treated with TMT was limited to the first cycle (3 months) spent in these recurrence health states. Following the first cycle after progression, the utility of remaining in that health state was presumed to be equivalent to that of surveillance after TMT.
- Cancer-specific mortality was assumed to occur only after progression to metastatic disease and was not allowed in non-progression health states (apart from 90-day cystectomy mortality probabilities).
- Long-term toxicities from RC and TMT were allowed to occur more than once. Disutility and costs were incurred during the cycle that the patient experienced the toxicity but not beyond that.
- Infectious and stone related long-term toxicities were assumed to be equivalent between RC and TMT treatment arms and were, therefore, not included in the model.
- Secondary malignancy from prior radiotherapy was not included in the model given lack of evidence in this patient population.
- If a patient experienced TMT GU major toxicity, we assumed that 50% had a procedure to address the complication and 50% did not.
- We assumed that utility values among patients experience first and second progression to metastatic disease was equivalent.
- We assumed equal rates of neoadjuvant chemotherapy use between TMT and RC treatment arms and so this was not included in the model.
- Surveillance protocols included within the model were based on NCCN guidelines for post-RC and post-TMT management. We assumed the following surveillance protocol based on NCCN guidelines.[1]
- We assumed diagnosis and workup of progression included two transurethral resections. Cost was derived from a weighted average assuming 50% of these resections were for 2-5cm tumors and 50% were >5cm tumors using CPT code 52240 and 52235.
- We assumed all NMIBC recurrences/progressions were treated with two transurethral resections followed by induction BCG without maintenance BCG. Costs for transurethral resections were derived from a weighted average assuming 50% of these resections were for 2-5cm tumors and 50% were >5cm tumors using CPT code 52240 and 52235.
- Due to the inability to accurately account for potential long-term genitourinary toxicities form TMT and additional toxicity from BCG, BCG toxicity was not included in the model.
- We assumed all progressions after BCG were treated with cystectomy regardless of NMIBC/MIBC status.
- We assumed all progressions to non-metastatic MIBC (unresponsive to TMT) were treated with salvage cystectomy.
- We assumed short-term toxicities from TMT could not occur following salvage cystectomy. GI-related long-term toxicities were assumed to be possible after salvage cystectomy, while GU-related toxicities were not.
- We assumed the progression probability after salvage cystectomy to be equal to that after primary cystectomy.[2]
- Short-term toxicities from treatments were no longer allowed to accrue once patients moved to a new health state with a different treatment. For example, toxicity from gemcitabine/cisplatin was no longer allowed after a 2$^{nd}$ progression treated with pembrolizumab.
- Systemic therapy short-term toxicities were allowed to accrue more than once while the patient remained in that progression health state.
- We assumed the risk of late complications with salvage cystectomy was twice that of non-salvage cystectomy. This assumption was varied in sensitivity analyses using a multiplier variable.[3]
- We assumed the risk of short-term toxicities after salvage RC was 1.3 times that of non-salvage RC. Given the higher base case probabilities for these toxicities we were not able to vary the multiplier higher as this would result in probabilities greater than 1. However, we were able to vary the multiplier lower to assume equal likelihood of short-term toxicities.[3]
- We assumed the probability of progression to either metastasis, MIBC, or NMIBC after five years was zero.[2]

© 2025 Joyce DD et al. *JAMA Network Open*.

- The mortality of second metastatic progression was assumed to be the same as mortality after first metastatic progression.

© 2025 Joyce DD et al. *JAMA Network Open*.

**eTable 1. Probabilities.** Transition probabilities for patients with MIBC undergoing TMT and RC and the corresponding reference used to inform these values. Ranges for each value represent the variance of each variable in sensitivity analyses.

| Health State | Probability | Range | Source |
|---|---|---|---|
| **TMT** | | | |
| **Progression (Metastatic)** | 0.26 | 0-0.38 | 4 |
| **Progression (MIBC) - Salvage Cystectomy** | 0.13 | 0-0.26 | 4 |
| **Progression (NMIBC)** | 0.205 | 0-0.34 | 4 |
| **Overall Mortality** | 0.30 | 0-0.50 | 4 |
| **Cancer-specific Mortality** | 0.17 | 0-0.29 | 4 |
| **Long-Term Toxicities** | | | |
| ***Minor*** | | | |
| GI (Diarrhea) | 0.019 | 0-0.15 | 5 |
| GU (Frequency/Intermittent GH) | 0.096 | 0-0.15 | 5 |
| ***Major*** | | | |
| GI (SBR) | 0.019 | 0-0.15 | 5 |
| GU (Severe Frequency/Dysuria/GH) | 0.057 | 0-0.15 | 5 |
| **Short-Term Toxicities** | | | |
| Mild (Cystitis/Dysuria) | 0.412 | 0-0.50 | 6 |
| Severe (Diarrhea) | 0.176 | 0-0.25 | 6 |
| **RC** | | | |
| **Progression (Metastatic)** | 0.26 | 0-0.38 | 4 |
| **Acute Complication** | | | 7 |
| Minor | 0.47 | 0-0.55 | 7 |
| Major | 0.19 | 0-0.35 | 7 |
| **1st line Treatment Toxicity (Gemcitabine/Cisplatin)** | | | |
| Minor (Nausea/Vomiting) | 0.22 | 0-0.30 | 8 |
| Major (Neutropenic Fever) | 0.299 | 0-0.35 | 8 |
| **2nd Line Treatment Toxicity (Pembrolizumab)** | | | |
| Minor (Diarrhea) | 0.631 | 0-0.70 | 9 |
| Major (Neutropenic fever) | 0.126 | 0-0.15 | 9 |
| **Progression After 1st Line Treatment (For Metastatic Disease)** | 0.9 | 0-0.97 | 8 |
| **Chronic Complication** | | | |
| Bowel | 0.008 | 0-0.14 | 10 |
| Stomal (Hernia Repair) | 0.008 | 0-0.14 | 10 |
| Ureteral Stricture | 0.008 | 0-0.14 | 10 |
| **Cystectomy Death** | 0.03 | 0-0.10 | 7 |
| **Cancer-Specific Mortality** | 0.17 | 0-0.29 | 4 |

TMT: trimodal therapy, RC: radical cystectomy, MIBC: muscle-invasive bladder cancer, NMIBC: non-muscle-invasive bladder cancer

**eTable 2. Costs.** Costs incurred within each possible health state for patients with MIBC undergoing TMT and RC and the corresponding billing code used to inform these values. Ranges for each value represent the variance of each variable in sensitivity analyses.

| Health State | CPT | DRG | HCPCS | Total COST | Range |
|---|---|---|---|---|---|
| **TMT** | 52235, 52240, 00840, 99205, 36569, 96413, 96415, 77263, 77014, 77300, 77334, 77290, 77295, 77280, 99205, 77263, 77300, 77334, 77290, 77280, 77334, 77295, 77336, 77427, 77412, 77417 | | J9060 | **$41,777.13** | **$0 - $62,665** |
| **Surveillance** | 99212, 52000, 74178, 71260, 88147, 80076, 85027, 80053 | | | $997.33 | $0 - $1,496 |
| **Long-Term Toxicities** | | | | | |
| **Minor** | | | | | |
| GI (Diarrhea) | 99205 | | | $197.06 | $0 - $1,000 |
| GU (Frequency/Intermittent GH) | 63739 | | | $262.50 | $0 - $500 |
| **Major** | | | | | |
| GI (SBR) | 44120 | 331 | | $16,317.87 | $0 - $24,477 |
| GU (Severe Frequency/Dysuria/GH) | | 695, 696 | | $3,847.82 | $0 - $10,000 |
| **Short-term toxicities** | | | | | |
| Mild (Cystitis/Dysuria) | 27845 | | | $262.50 | $0 - $394 |
| Severe (Diarrhea) | | 392 | | $7,356.18 | $0 - $11,034 |
| **RC** | 99205, 51550 | 655, 654 | | **$19,923.01** | **$0 - $50,000** |
| **Surveillance** | 99212, 74178, 71260, 88147, 80076, 85027, 80053 | | | $637.33 | $0 - $1,000 |
| **Acute complication** | | | | | |
| Minor | | 707, 708 | | $7,243.16 | $0 - $11,000 |
| Major | | 707, 708 | | $7,243.16 | $0 - $11,000 |
| **Chronic Complication** | | | | | |
| Bowel | 44120 | 331 | | $16,317.87 | $0 - $24,477 |
| Stomal (Hernia Repair) | 50727 | | | $3,018.54 | $0 - $4,527 |
| Ureteral Stricture | 50693 | 661 | | $6,592.96 | $0 - $9,890 |
| **Progression** | | | | | |
| **Progression (Metastatic) - Gemcitabine/Cisplatin** | | | J9201, J9060 | $2,427.25 | $0 - $3,640 |
| **1st Line Treatment Toxicity (Gemcitabine/Cisplatin)** | | | | | |

© 2025 Joyce DD et al. *JAMA Network Open*.

| Health State | CPT | DRG | HCPCS | Total COST | Range |
|---|---|---|---|---|---|
| Minor (Nausea/Vomiting) | 99205 | | | $186.90 | $0 - $300 |
| Major (Neutropenic Fever) | | 809 | | $11,745.74 | $0 - $18,000 |
| **Health State** | **CPT** | **DRG** | **HCPCS** | **Total COST** | **Range** |
| **2nd Line Treatment (Metastatic) - Pembrolizumab** | | | J9271 | $43,130.84 | $0 - $100,000 |
| **2nd Line Treatment Toxicity (Pembrolizumab)** | | | | | |
| Minor (Diarrhea) | 99205 | | | $211.12 | $0 - $400 |
| Major (Neutropenic Fever) | | 809 | | $11,745.74 | $0 - $23,000 |
| **Progression (MIBC) - Salvage Cystectomy without Complications** | 51550 | 708 | | $19,923.01 | $0 - $50,000 |
| **Progression (MIBC) - Salvage Cystectomy with Complications** | | 707 | | $53,483.23 | $0 - $61,000 |
| **Progression (NMIBC)** | 52235, 52240, 00840 | | J9030 | $9,441.08 | $0 - $14,162 |

TMT: trimodal therapy, RC: radical cystectomy, MIBC: muscle-invasive bladder cancer, NMIBC: non-muscle-invasive bladder cancer, SBR: small bowel resection

© 2025 Joyce DD et al. *JAMA Network Open*.

**eTable 3. Utilities.** Health state utility values for patients with MIBC undergoing TMT and RC and the corresponding reference used to inform these values. Ranges for each value represent the variance of each variable in sensitivity analyses. Tolls for each value represent the time course over which each utility was experienced.

| | Utility | Toll | Range | Source |
|---|---|---|---|---|
| **TMT** | 0.64 | 90 days | 0-1 | 11 |
| Surveillance | 0.98 | Lifetime | 0-1 | 12 |
| **Long-Term Toxicities** | | | | |
| ***Minor*** | | | | |
| GI (Diarrhea) | 0.8 | 2 weeks | 0-1 | 11 |
| GU (Frequency/Intermittent GH) | 0.9 | 3 months | 0-1 | 13 |
| ***Major*** | | | | |
| GI (SBR) | 0.76 | 3 months | 0-1 | 14 |
| GU (Severe Frequency/Dysuria/GH) | 0.77 | 3 months | 0-1 | 15 |
| **Short-term toxicities** | | | | |
| Mild (Cystitis/Dysuria) | 0.9 | 90 days | 0-1 | 13 |
| Severe (Diarrhea) | 0.521 | 2 weeks | 0-1 | 16 |
| **RC** | 0.8 | 90 days | 0-1 | 11 |
| Surveillance | 0.98 | Lifetime | 0-1 | 12 |
| **Acute complication** | | | | |
| Minor | 0.77 | 2 weeks | 0-1 | 11 |
| Major | 0.73 | 2 weeks | 0-1 | 11 |
| **Chronic Complication** | | | | |
| Bowel (SBR) | 0.76 | 90 days | 0-1 | 14 |
| Stomal (Hernia Repair) | 0.76 | 90 days | 0-1 | 11,14 |
| Ureteral Stricture | 0.76 | 90 days | 0-1 | 11,14 |
| **Progression** | | | | |
| Progression (MIBC) - Salvage Cystectomy | 0.8 | 90 days | 0-1 | 11 |
| Progression (NMIBC) - TURBT | 0.9 | 2 weeks | 0-1 | 11 |
| Progression (Metastatic) | 0.62 | Lifetime | 0-1 | 11 |
| **1st line Treatment Toxicity (Gemcitabine/Cisplatin)** | | | | |
| Minor (Nausea/Vomiting) | 0.8 | 2 weeks | 0-1 | 11 |
| Major (Neutropenic Fever) | 0.47 | 2 weeks | 0-1 | 17 |
| **2nd Line Treatment Toxicity (Pembrolizumab)** | | | | |
| Minor (Diarrhea) | 0.8 | 2 weeks | 0-1 | 11 |
| Major (Neutropenic fever) | 0.47 | 2 weeks | 0-1 | 17 |

TMT: trimodal therapy, RC: radical cystectomy, MIBC: muscle-invasive bladder cancer, NMIBC: non-muscle-invasive bladder cancer, TURBT: transurethral resection of bladder tumor, GH: gross hematuria, SBR: small bowel resection.

© 2025 Joyce DD et al. *JAMA Network Open*.

**eFigure 1. State transition diagram.** Ellipses indicate each potential health state within the microsimulation model. Arrows indicate the direction of transitions from one health state to another. Progression to NMIBC and MIBC were only allowed in the trimodal therapy treatment arm. Toxicity transition probabilities were specific to each treatment option.

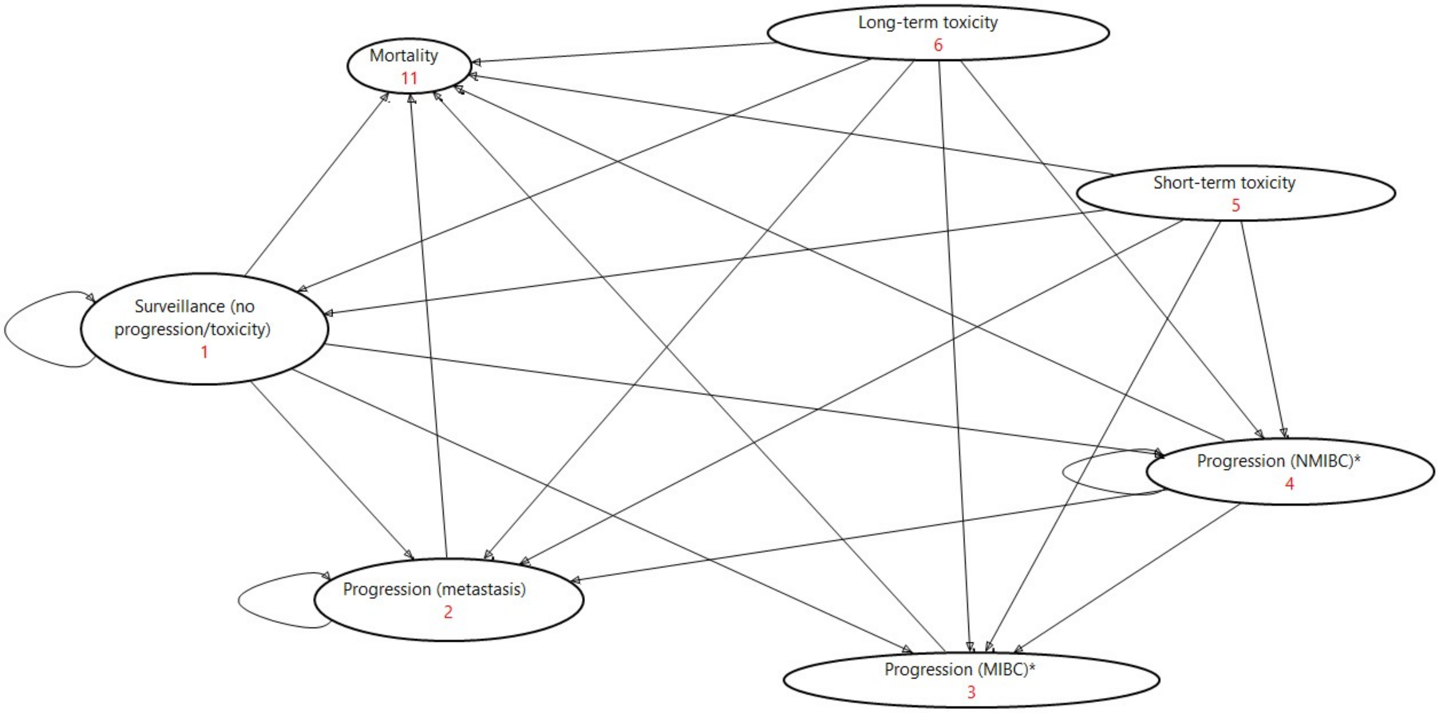


MIBC: muscle-invasive bladder cancer; NMIBC: non-muscle-invasive bladder cancer. *Denotes health states not present in the radical cystectomy treatment arm.

© 2025 Joyce DD et al. *JAMA Network Open*.

**eFigure 2. Incremental cost and effectiveness histograms.** Distribution of comparative outcomes over 100,000 microsimulations at a 5-year time horizon. A: Histogram of incremental costs. B: Histogram of incremental effectiveness.

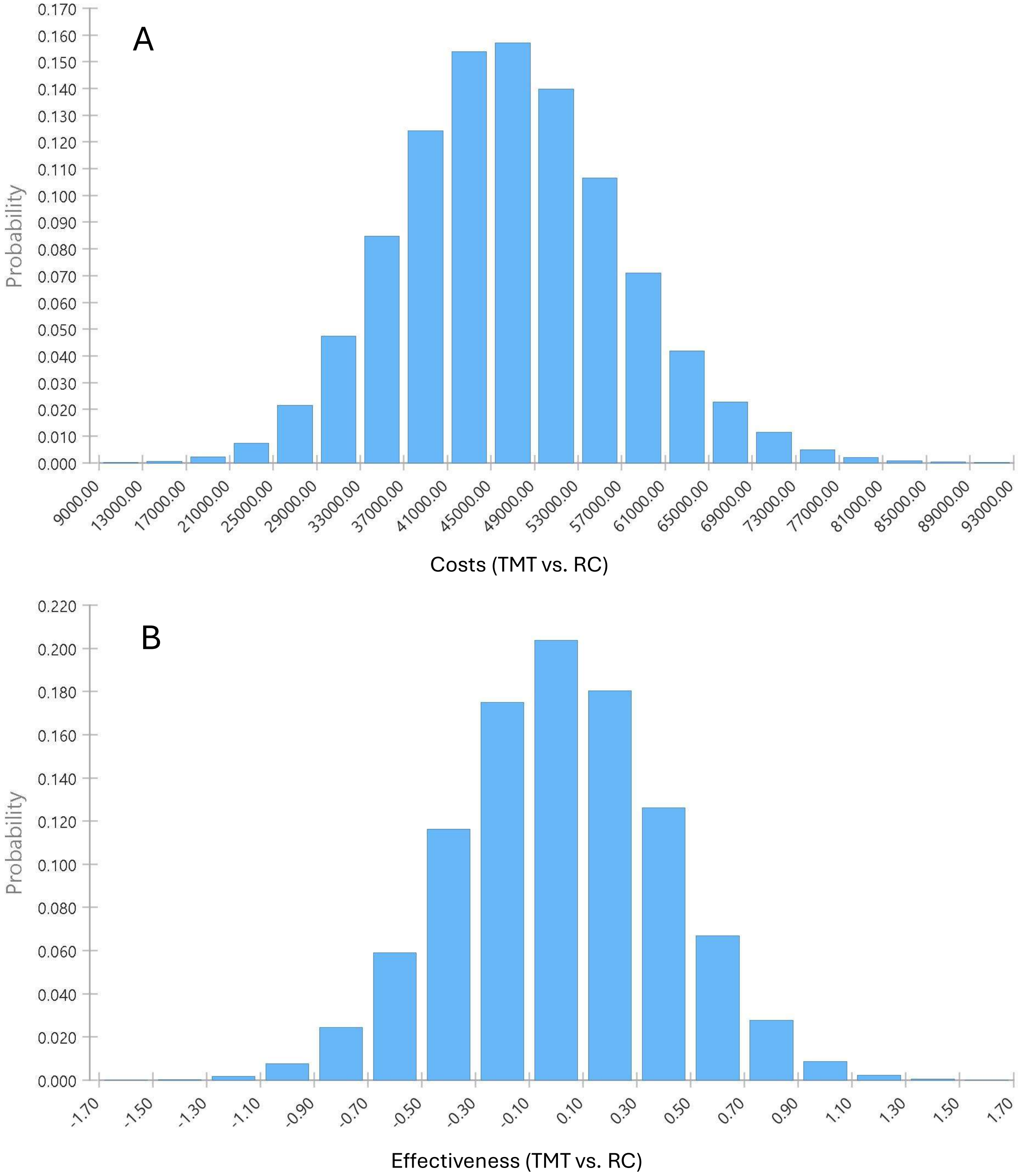


© 2025 Joyce DD et al. *JAMA Network Open*.

**eFigure 3. Incremental cost and effectiveness histograms.** Distribution of comparative outcomes over 100,000 microsimulations at a 10-year time horizon. A: Histogram of incremental costs. B: histogram of incremental effectiveness.

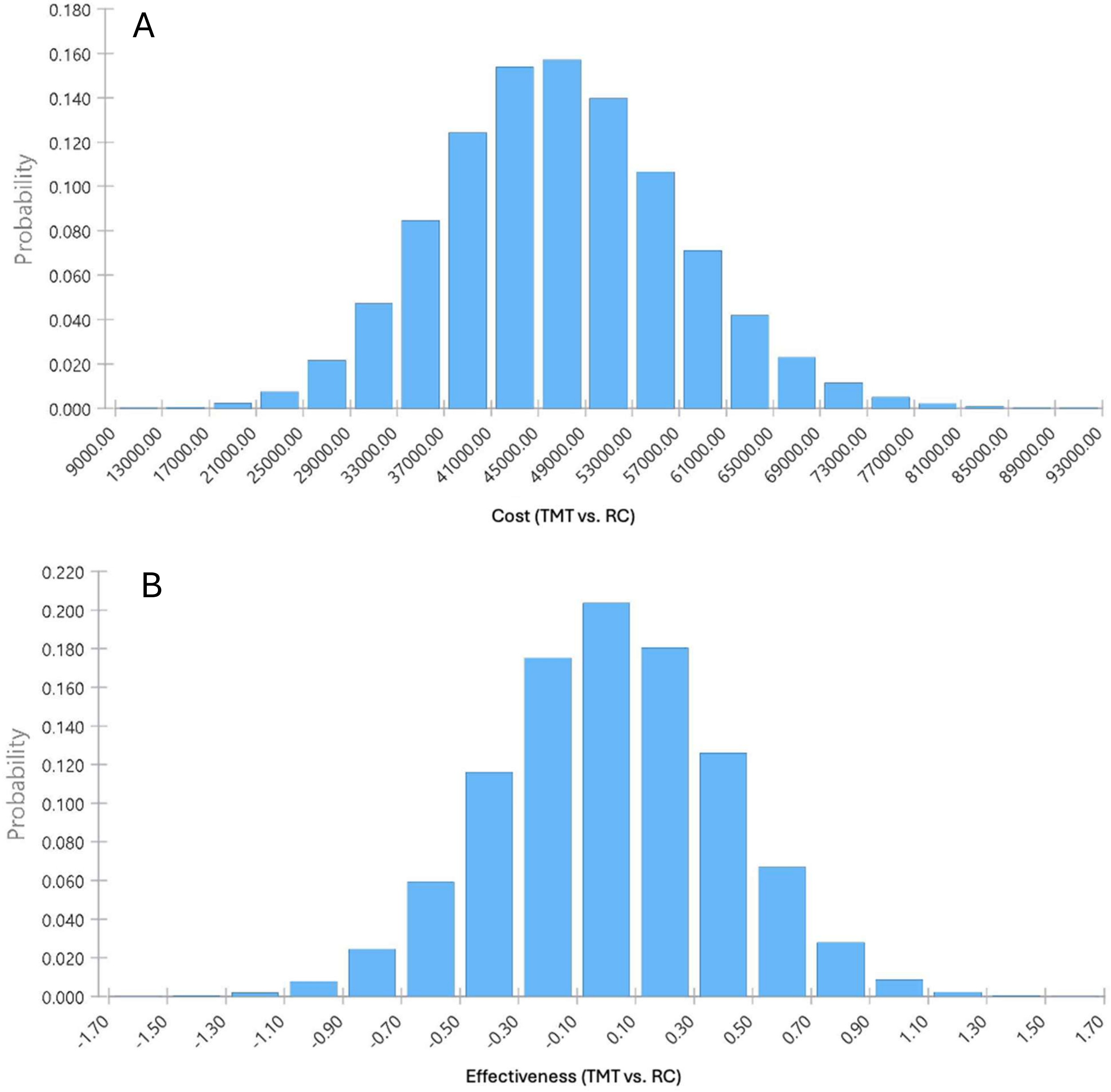


© 2025 Joyce DD et al. *JAMA Network Open*.

**eReferences**


1. Referenced with permission from the NCCN Clinical Practice Guidelines in Oncology (NCCN Guidelines®) for Bladder Cancer V.4.2024. © National Comprehensive Cancer Network, Inc. 2024. All rights reserved. Accessed [Sep 20, 2024]. To view the most recent and complete version of the guideline, go online to NCCN.org.
2. Zlotta AR, Ballas LK, Niemierko A, et al. Radical cystectomy versus trimodality therapy for muscle-invasive bladder cancer: a multi-institutional propensity score matched and weighted analysis. *Lancet Oncol*. Jun 2023;24(6):669-681. doi:10.1016/S1470-2045(23)00170-5
3. Pieretti A, Krasnow R, Drumm M, et al. Complications and Outcomes of Salvage Cystectomy after Trimodality Therapy. *J Urol*. Jul 2021;206(1):29-36. doi:10.1097/JU.0000000000001696
4. Zlotta AR, Ballas LK, Niemierko A, et al. Radical cystectomy versus trimodality therapy for muscle-invasive bladder cancer: a multi-institutional propensity score matched and weighted analysis. *Lancet Oncol*. Jun 2023;24(6):669-681. doi:10.1016/S1470-2045(23)00170-5
5. Giacalone NJ, Shipley WU, Clayman RH, et al. Long-term Outcomes After Bladder-preserving Tri-modality Therapy for Patients with Muscle-invasive Bladder Cancer: An Updated Analysis of the Massachusetts General Hospital Experience. *Eur Urol*. Jun 2017;71(6):952-960. doi:10.1016/j.eururo.2016.12.020
6. Tunio MA, Hashmi A, Qayyum A, Mohsin R, Zaeem A. Whole-pelvis or bladder-only chemoradiation for lymph node-negative invasive bladder cancer: single-institution experience. *Int J Radiat Oncol Biol Phys*. Mar 1 2012;82(3):e457-62. doi:10.1016/j.ijrobp.2011.05.051
7. Parekh DJ, Reis IM, Castle EP, et al. Robot-assisted radical cystectomy versus open radical cystectomy in patients with bladder cancer (RAZOR): an open-label, randomised, phase 3, non-inferiority trial. *Lancet*. Jun 23 2018;391(10139):2525-2536. doi:Doi 10.1016/S0140-6736(18)30996-6
8. von der Maase H, Hansen SW, Roberts JT, et al. Gemcitabine and cisplatin versus methotrexate, vinblastine, doxorubicin, and cisplatin in advanced or metastatic bladder cancer: results of a large, randomized, multinational, multicenter, phase III study. *J Clin Oncol*. Sep 2000;18(17):3068-77. doi:10.1200/JCO.2000.18.17.3068
9. Bellmunt J, de Wit R, Vaughn DJ, et al. Pembrolizumab as Second-Line Therapy for Advanced Urothelial Carcinoma. *New Engl J Med*. Mar 16 2017;376(11):1015-1026. doi:10.1056/NEJMoa1613683
10. Shimko MS, Tollefson MK, Umbreit EC, Farmer SA, Blute ML, Frank I. Long-term complications of conduit urinary diversion. *J Urol*. Feb 2011;185(2):562-7. doi:10.1016/j.juro.2010.09.096
11. Kulkarni GS, Alibhai SM, Finelli A, et al. Cost-effectiveness analysis of immediate radical cystectomy versus intravesical Bacillus Calmette-Guerin therapy for high-risk, high-grade (T1G3) bladder cancer. *Cancer*. Dec 1 2009;115(23):5450-9. doi:10.1002/cncr.24634
12. Hao S, Karlsson A, Heintz E, Elfstrom KM, Nordstrom T, Clements M. Cost-Effectiveness of Magnetic Resonance Imaging in Prostate Cancer Screening: A Microsimulation Study. *Value Health*. Dec 2021;24(12):1763-1772. doi:10.1016/j.jval.2021.06.001
13. Jewell EL, Smrtka M, Broadwater G, et al. Utility scores and treatment preferences for clinical early-stage cervical cancer. *Value Health*. Jun 2011;14(4):582-6. doi:10.1016/j.jval.2010.11.017
14. Li K, Cannon JGD, Jiang SY, et al. Diagnostic staging laparoscopy in gastric cancer treatment: A cost-effectiveness analysis. *J Surg Oncol*. May 2018;117(6):1288-1296. doi:10.1002/jso.24942
15. Freemantle N, Khalaf K, Loveman C, et al. OnabotulinumtoxinA in the treatment of overactive bladder: a cost-effectiveness analysis versus best supportive care in England and Wales. *Eur J Health Econ*. Sep 2016;17(7):911-21. doi:10.1007/s10198-015-0737-2
16. Riesco-Martinez MC, Berry SR, Ko YJ, et al. Cost-Effectiveness Analysis of Different Sequences of the Use of Epidermal Growth Factor Receptor Inhibitors for Wild-Type KRAS Unresectable Metastatic Colorectal Cancer. *J Oncol Pract*. Jun 2016;12(6):e710-23. doi:10.1200/JOP.2015.008730
17. Tumeh JW, Shenoy PJ, Moore SG, Kauh J, Flowers C. A Markov model assessing the effectiveness and cost-effectiveness of FOLFOX compared with FOLFIRI for the initial treatment of metastatic colorectal cancer. *Am J Clin Oncol*. Feb 2009;32(1):49-55. doi:10.1097/COC.0b013e31817c6a4d


© 2025 Joyce DD et al. *JAMA Network Open*.
