# Supplementary material for: Cost-Effectiveness of Trimodal Therapy and Radical Cystectomy for Muscle-Invasive Bladder Cancer
Source: JAMA Netw Open. 2025 Jun 23;8(6):e2517056. doi: 10.1001/jamanetworkopen.2025.17056 (PMC12186510; doi:10.1001/jamanetworkopen.2025.17056)
Supplement: Supplement 2. — Data Sharing Statement [file jamanetwopen-e2517056-s002.pdf]

Data Sharing Statement

Joyce. Cost-Effectiveness of Trimodal Therapy and Radical Cystectomy for Muscle-Invasive Bladder Cancer. *JAMA Netw Open*. Published June 23, 2025.
doi:10.1001/jamanetworkopen.2025.17056

Data
Data available: Yes
Data types: Data (not involving human participants)
How to access data: All data is provided within the submitted manuscript.
When available: With publication

Supporting Documents
Document types: None

Additional Information
Who can access the data: Anyone requesting the data
Types of analyses: for any purpose
Mechanisms of data availability: with investigator support
Any additional restrictions: none
